# Subcritical Water Extraction of Valuable Metals from Spent Lithium-Ion Batteries

**DOI:** 10.3390/molecules25092166

**Published:** 2020-05-06

**Authors:** Jenni Lie, Stefani Tanda, Jhy-Chern Liu

**Affiliations:** Department of Chemical Engineering, National Taiwan University of Science and Technology, 43 Keelung Road, Section 4, Taipei 106, Taiwan; d10706801@mail.ntust.edu.tw (J.L.); stefanitanda@yahoo.co.id (S.T.)

**Keywords:** ascorbic acid, hydrochloric acid (HCl), leaching, Li-ion batteries (LIBs), subcritical water extraction (SWE), waste

## Abstract

The leaching of valuable metals (Co, Li, and Mn) from spent lithium-ion batteries (LIBs) was studied using subcritical water extraction (SWE). Two types of leaching agents, hydrochloric acid (HCl) and ascorbic acid, were used, and the effects of acid concentration and temperature were investigated. Leaching efficiency of metals increased with increasing acid concentration and temperature. Ascorbic acid performed better than HCl, which was attributed to ascorbic acid’s dual functions as an acidic leaching agent and a reducing agent that facilitates leaching reactions, while HCl mainly provides acidity. The chemical analysis of leaching residue by X-ray photoelectron spectroscopy (XPS) revealed that Co(III) oxide could be totally leached out in ascorbic acid but not in HCl. More than 95% of Co, Li, and Mn were leached out from spent LIBs’ cathode powder by SWE using 0.2 M of ascorbic acid within 30 min at 100 °C, initial pressure of 10 bar, and solid-to-liquid ratio of 10 g/L. The application of SWE with a mild concentration of ascorbic acid at 100 °C could be an alternative process for the recovery of valuable metal in spent LIBs. The process has the advantages of rapid reaction rate and energy efficiency that may benefit development of a circular economy.

## 1. Introduction

Lithium-ion batteries (LIBs) are the main choice as the electrochemical power source for most portable electronic devices and electric vehicles (EVs). The rapid development of and high demand for electronic devices and EVs have caused the rapid increase of both the number of spent LIBs as well as the demand for metal resources, especially lithium (Li) and cobalt (Co). More than one million EVs worldwide were sold in 2017, which is estimated to generate around 250,000 tons of battery waste when they reach their end of life [1]. Besides which, the organic electrolytes and heavy metals in spent LIBs are toxic, which could be a risk for human health and the environment if the spent LIBs are disposed of in landfill [2,3]. Meanwhile, a huge gap between market supply and demand leads to an increase in the price of critical metals, especially for Li and Co [4]. The spent LIBs have gained a lot of attention as an urban mining source of valuable metals such as Li, Co, Ni, Al, and Mn. The recovery of valuable metals from spent LIBs is essential to avoid a negative environmental impact and to ensure the safe supply of the corresponding materials [5].

Numerous studies on the recycling of spent LIBs using hydrometallurgy, pyrometallurgy, and biometallurgy have been reported. Pyrometallurgical processes have been adopted and commercialized for valuable metal recycling [6], however, high energy consumption and toxic gas emissions are the main problems of these processes. Hydrometallurgical methods are under study for recycling of spent LIBs due to the mild reaction conditions, environmental friendliness (non-toxic emissions), and high recovery efficiency of valuable metals [7]. They have been developed for metals extraction from spent LIBs using inorganic acids such as HCl and H_2_SO_4_. However, organic acid is known as an eco-friendly lixiviant, which is biodegradable and has non-toxic gas emissions [8,9]. Leaching of lithium cobalt oxide (LiCoO_2_) is difficult due to the chemical bonds between cobalt and oxygen, which are extremely strong [10,11]. In the case of leaching with ascorbic acid (C_6_H_8_O_6_), the waste LiCoO_2_ is dissolved and forms the soluble compound C_6_H_6_O_6_Li_2_, while Co(III) in LiCoO_2_ are further reduced to a soluble Co(II) by ascorbic acid. In the meantime, C_6_H_8_O_6_ is oxidized to dehydroascorbic acid (C_6_H_6_O_6_) [12]. Subcritical water is one of the energy intensification processes; it is achieved by applying pressurized (10 to 60 bar) water in the temperature range of 100–374 °C that leads to enhanced mass transfer and accelerated chemical reaction [13]. Liu and Zhang [14] reported that more than 95% of Co and Li were recovered from spent LIBs using polyvinyl chloride (PVC) in subcritical water extraction (SWE) under optimum condition of 350 °C for 30 min. A more recent study by Nshizirungu et al. [15] used waste chlorinated PVC (CPVC) as the source of HCl for Li and Co leaching from spent LIBs in a hydrothermal subcritical water process at 250 °C for 60 min, and higher than 97%, leaching efficiency of both metals was found. Although waste CPVC could be degraded, the process required high energy to decompose the PVC and then to release Cl^−^ ions [16]. Therefore, the leaching of valuable metals (Li, Co, and Mn) from spent LIBs using HCl and ascorbic acid under lower temperatures (100 to 150 °C) by SWE was investigated in the current study.

To further assess the leaching mechanisms, the leaching residues of HCl and ascorbic acid were analyzed by X-ray photoelectron spectroscopy (XPS). This study aimed to examine the leaching of valuable metals from spent LIBs using an SWE process and to explore redox reactions at the spent LIBs’ surfaces.

## 2. Results

### 2.1. Effect of Acid Concentration

**Figure 1:**
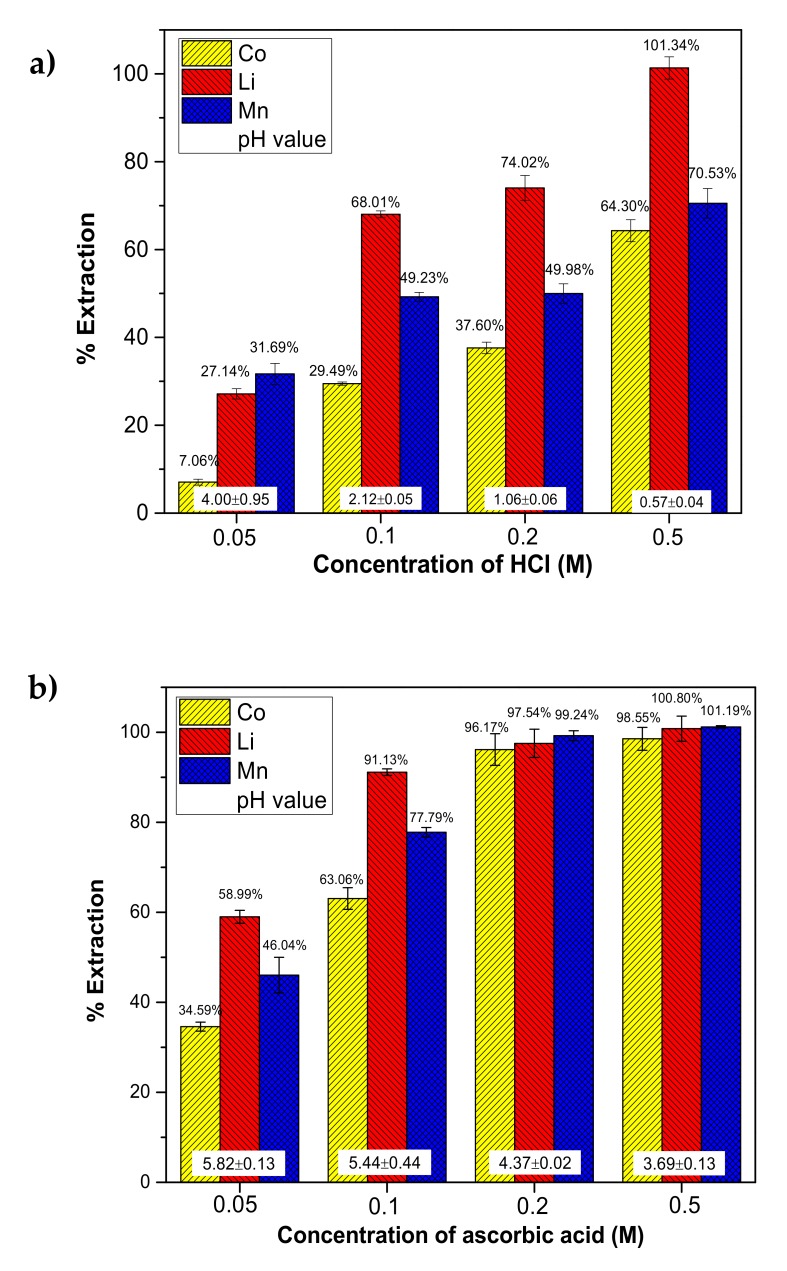
Effect of concentration on subcritical water extraction (SWE) at 100 °C, 10 bar, and solid-to-liquid (S/L) ratio of 10 g/L for 30 min using (**a**) HCl and (**b**) ascorbic acid.

The effect of acid concentration on SWE was studied using 0.05, 0.1, 0.2, and 0.5 M of leaching agent solutions at 100 °C for 30 min, solid-to-liquid (S/L) ratio of 10 g/L, 300 rpm stirring speed, and initial pressure of 10 bar. Figure 1 shows that the concentration of the leaching agent had a significant effect on leaching efficiency of Co, Li, and Mn. The leaching efficiency of Co, Li, and Mn increased from 7.06% to 29.49%, 27.14% to 68.01%, and 31.69% to 49.23%, respectively, as the concentration of HCl increased from 0.05 M to 0.1 M, and further increased to 64.30%, 101.34%, and 70.53% at 0.5 M (Figure 1a). A similar trend was found when using ascorbic acid as shown in Figure 1b. The leaching efficiency increased from 34.59% to 96.17% for Co, from 58.99% to 97.54% for Li, and from 46.04% to 99.24% for Mn by increasing the concentration of ascorbic acid from 0.05 M to 0.2 M. The excess leaching efficiency (>100%) as shown in some data may be due to non-uniform metal content in the spent LIBs. Ascorbic acid resulted in a higher leaching efficiency than HCl as a leaching agent for Co, Li, and Mn in spent LIBs, which may be attributed to its high reducibility. The deionization of ascorbic acid (H_2_Asc) is depending on the solution pH, H_2_Asc will form ascorbate ion (HAsc^−^) and ascorbate dianion (Asc^2−^) which is easily oxidized to dehydroascorbic acid (DAsc) [17]. The pKa values of ascorbic acid are pKa_1_ of 4.10 and pKa_2_ of 11.6 [12]. The pH of leaching solution revealed that the ascorbic acid existed in the solution as HAsc^−^ at concentrations 0.05 to 0.2 M (pH > 4.10), and as H_2_Asc at 0.5 M (pH < 4.10). Table 1 shows that both HCl and ascorbic acid could be used as a reducing agent, however, the reduction potentials of HAsc^−^ and H_2_Asc are much lower than that of Cl^−^, and 1.78 V and 1.73 V of overall potential was found in the reactions with Co(III), which is higher than 1.24 V when Cl^−^ was involved. When ascorbic acid is oxidized, two electrons are released. Those electrons were used to reduce Co(III) in spent LIBs to Co(II), which is known to be more soluble in solution [18,19]. The addition of other reducing agents, such as hydrogen peroxide (H_2_O_2_), sodium thiosulfate (Na_2_S_2_O_3_), and glucose (C_6_H_12_O_6_) can enhance the dissolution of cobalt oxide [20,21]. The higher potential of overall reactions of ascorbic acid compounds (H_2_Asc and HAsc^−^) with cobalt (III) represent a higher driving force of the redox reaction.

The stoichiometric concentrations of HCl and ascorbic acid to leach Li, Co, and Mn from 10 g/L of spent LIBs were 0.4 M and 0.2 M. However, when using 0.5 M of HCl, S/L of 10 g/L, 100 °C for 30 min resulted in leaching of only 64.30% of Co, 101.34% of Li, and 70.53% of Mn. On the other hand, higher than 95% leaching efficiency of Co, Li, and Mn, respectively, was found when using 0.2 M of ascorbic acid. These results confirmed that the HCl only provided acidity and limited the reducing capability to induce the leaching of Co, Li, and Mn in spent LIBs. While in the leaching process using ascorbic acid, Li was first dissolved by forming C_6_H_6_O_6_Li_2_, and Co(III)_,_ Co(IV), and Mn(IV) in spent LIBs were reduced to the more soluble Co(II) and Mn(II), while ascorbic acid (C_6_H_8_O_6_) was oxidized to dehydroascorbic acid (C_6_H_6_O_6_) [12]. The high temperature and pressure adopted in SWE enhanced solubility and mass transfer, and resulted in a more efficient leaching process with a lower concentration of leaching agent.

### 2.2. Effect of Temperature

**Figure 2:**
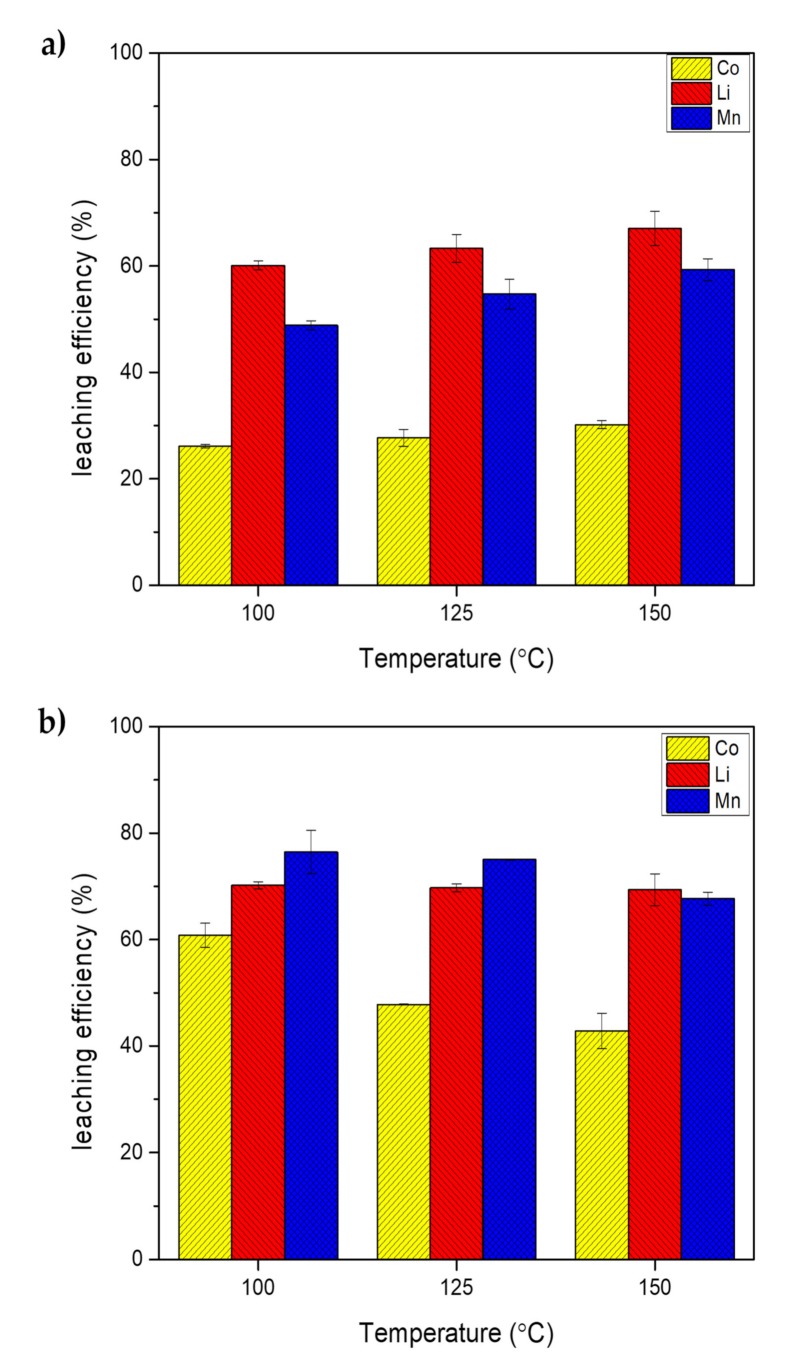
Effect of temperature on SWE at S/L of 10 g/L for 30 min using 0.1 M of (**a**) HCl and (**b**) ascorbic acid.

The effect of temperature on SWE was studied using 0.1 M of leaching agent, initial pressure of 10 bar, stirring speed of 300 rpm, solid-to-the liquid ratio of 10 g/L, for 30 min. As shown in Figure 2a, the leaching efficiency of Co, Li, and Mn slightly increased from 26.14% to 27.9%, from 60.11% to 63.31%, and from 48.85% to 54.73%, respectively, when temperature increased from 100 to 125 °C using HCl. At 150 °C, the leaching efficiency of Co, Li, and Mn rose to 30.19%, 67.05%, and 59.31%, respectively. This might be attributed to more dissociation of water into hydroxonium ions (H_3_O^+^) under subcritical conditions at a higher temperature, which improved the hydrolysis of valuable metals in the leaching process [15]. Figure 2b shows that leaching efficiency of Co, Li, and Mn decreased from 60.81% to 47.79%, from 70.21% to 69.70%, and from 76.45% to 75.03%, respectively, by increasing temperature from 100 to 125 °C when using ascorbic acid. The efficiency of Co, Li, and Mn became even lower at 42.81%, 69.33%, and 67.66%, respectively, at 150 °C. This was probably due to the decomposition of ascorbic acid in a hydrothermal condition. Subcritical water induces the decomposition of organic compounds into smaller fragments [14]. Karpushkin et al. [24] studied the hydrothermal transformations of 10% ascorbic acid at 160 °C for 2 to 24 h and the results indicated that the decomposition of ascorbic acid was accompanied by decarboxylation to form insoluble compounds and other gaseous products. Another investigation by Mogol and Gökmen [25] reported the degradation rate of ascorbic acid to intermediate products by hydrolysis was higher than that of ascorbic acid oxidation to dehydroascorbic acid as heating temperature increased to 140 °C. It is noted that even though effective leaching for Co, Li, and Mn from spent LIBs by SWE is possible using low concentrations of ascorbic acid, the temperature should be kept as low as possible. On the contrary, higher acid concentration and temperature are necessary when using HCl. However, ascorbic acid is more expensive than HCl, therefore a cost-effectiveness analysis is required in selecting the optimum conditions for the recycling process of spent LIBs.

### 2.3. Dissolution of Cobalt from Spent LIBs

**Figure 3:**
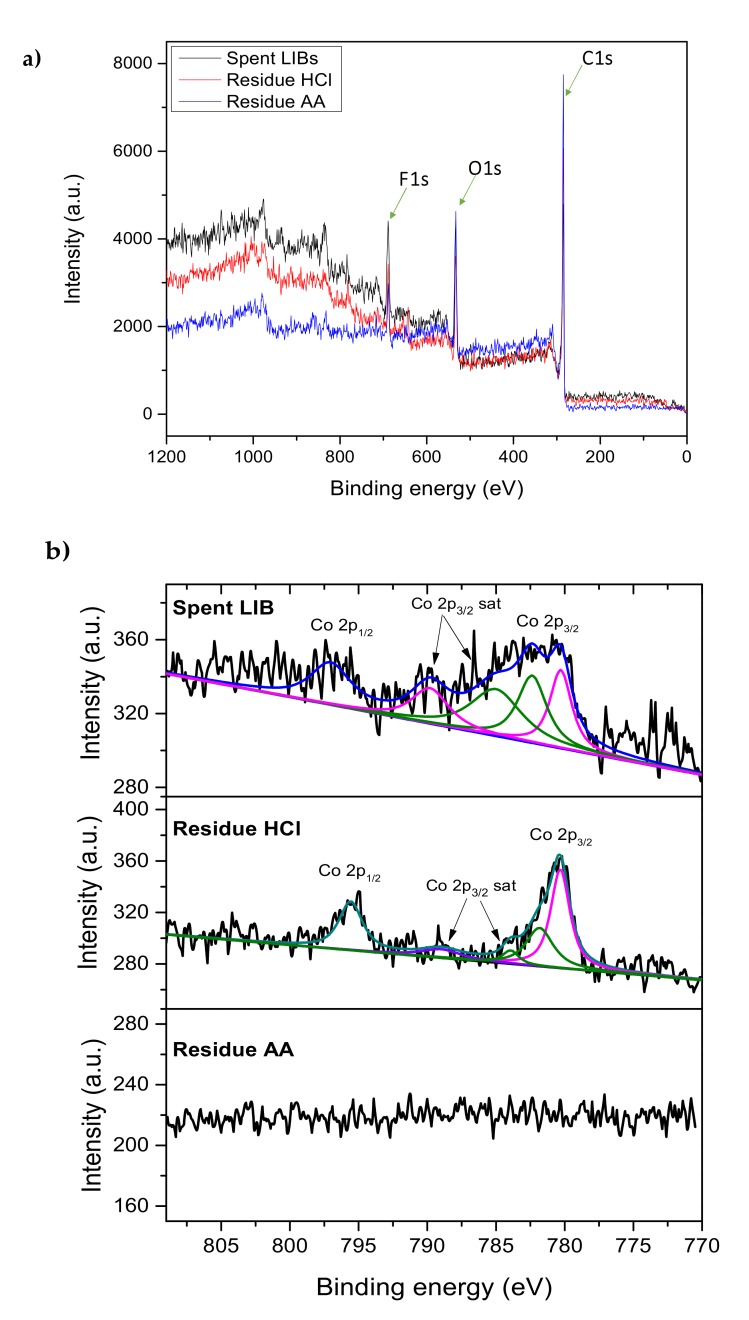
XPS result of spent LIBs and leaching residue using HCl and ascorbic acid (AA) in SWE (**a**) wide scan and (**b**) Co 2p peak.

As mentioned in the previous section, Co leaching is enhanced as Co(III) is reduced to a more soluble Co(II) compound. However, most published papers have only postulated that some reducing agents could be induce the reaction by standard redox potential [17,18,19,20]. To examine redox reactions occurring during SWE leaching at the spent LIBs’ surfaces, X-ray photoelectron spectroscopy (XPS) was used to assess the chemical composition of the solid before and after leaching in 0.5 M of HCl and 0.5 M of ascorbic acid using SWE at 100 °C for 30 min. The wide scan of the sample in Figure 3a shows the decreasing intensity of the XPS spectra of spent LIBs after being subjected to the leaching process using HCl and ascorbic acid (AA) due to the dissolution of the compounds. The highest peaks in XPS spectra of spent LIBs’ powder included C1s as carbon or graphite content of the anode powder in LIBs, O1s as oxygen content from the oxide compounds in LIBs, and F1s as the fluoride content in LIBs’ binders, mostly polyvinylidene fluoride (PVDF). Figure 3b shows that XPS spectra of the Co 2p peak of spent LIBs consisted of Co 2p_3/2_, Co 2p_3/2_ satellites, and Co 2p_1/2_. The corresponding binding energy (BE) and the compositions are illustrated in Table 2. The presence of Co in the LIBs’ cathode (LiCoO_2_) is predominantly in the form of Co(III). It is transformed into the inactive compounds (mostly Co_3_O_4_, followed by Co_2_O_3_ and CoO) in spent LIBs due to the reactions during migration of Co ions and due to the detrimental effect of the binder [26]. The ratio of Co(II) to Co(III) in spent LIBs was 1.141, implying that the concentration of Co(II) oxide was higher than Co(III) oxide. The satellite peaks of Co 2p_3/2_ were represented at 785 eV and 789.8 eV, corresponding to Co(II) and Co(III) excitation [27].

The peak area of Co(II) in the leaching residue using HCl became much smaller and the ratio of Co(II) to Co(III) decreased to 0.550. This confirmed that most of the Co(II) oxide in spent LIBs was dissolved in HCl but not for Co(III) oxide. The XPS results of cobalt demonstrated that dissolution of the Co(II) compound is more significant in acid solution than that of Co(III). A significant amount of Co(III) was still found in the leaching residue using 0.5 M HCl, while no Co 2p peak could be detected when using ascorbic acid. This was due to the complete dissolution of Co content in spent LIBs in the leaching process. These results were in agreement with the aforementioned redox potential prediction that ascorbic acid had a higher driving force than HCl to reduce Co(III) to Co(II).

The XRD spectra of leaching residue of HCl and ascorbic acid (Figure S7) showed that the leaching residue of HCl still contained LiCoO_2_, Li_2_CoMn_3_O_8_, and Co_3_O_4_, while the leaching residue of ascorbic acid mostly contained carbon of the anode powder, elemental Cu (COD 9011604) from the reduction Cu(II) by ascorbic acid [28,29], and the insoluble product of ascorbic acid hydrolysis, D-isoascorbic acid (PDF 32–1637).

### 2.4. Conventional Leaching

The experiments using a conventional leaching method were carried out using HCl and ascorbic acid at three different temperatures (25, 45, and 65 °C), different reaction times (1, 2, 4, 6, 8, 10, 20, 30, 60, 90, 120, 180, 240, 360, 480, 600, and 720 min), acid concentration of 0.5 M, and S/L of 10 g/L. The leaching efficiency of Co, Li, and Mn from cathode powder of spent LIBs at various temperatures are shown in Figures S1–S6. Lower leaching efficiency of Co, Li, and Mn was found compared with those of SWE. The leaching efficiency of Co, Li, and Mn using HCl at 65 °C for 4 h were 42.19%, 80.46%, and 54.13%, respectively, as compared with 96.36% of Co, 95.54% of Li, and 97.9% of Mn using ascorbic acid at 65 °C for 4 h min. In general, the conventional extraction required a much longer time to achieve comparable leaching efficiency of Li, Co, and Mn when compared with SWE. The activation energy of Co, Li, and Mn was determined by using the Arrhenius equation from conventional leaching kinetic study (Tables S1–S6), which fitted well with a pseudo-second-order kinetic model. Table S7 shows that the activation energy of Co, Li, and Mn in HCl are higher than in ascorbic acid. In addition, the activation energy of a diffusion-controlled process is usually below 40 kJ/mol, while for a chemically controlled reaction, the value is usually greater than 40 kJ/mol [29,30]. Therefore, both diffusion and chemical reaction processes were involved in the leaching of Co, Li, and Mn from spent LIBs using HCl and ascorbic acid.

## 3. Material and Methods

**Figure 4:**
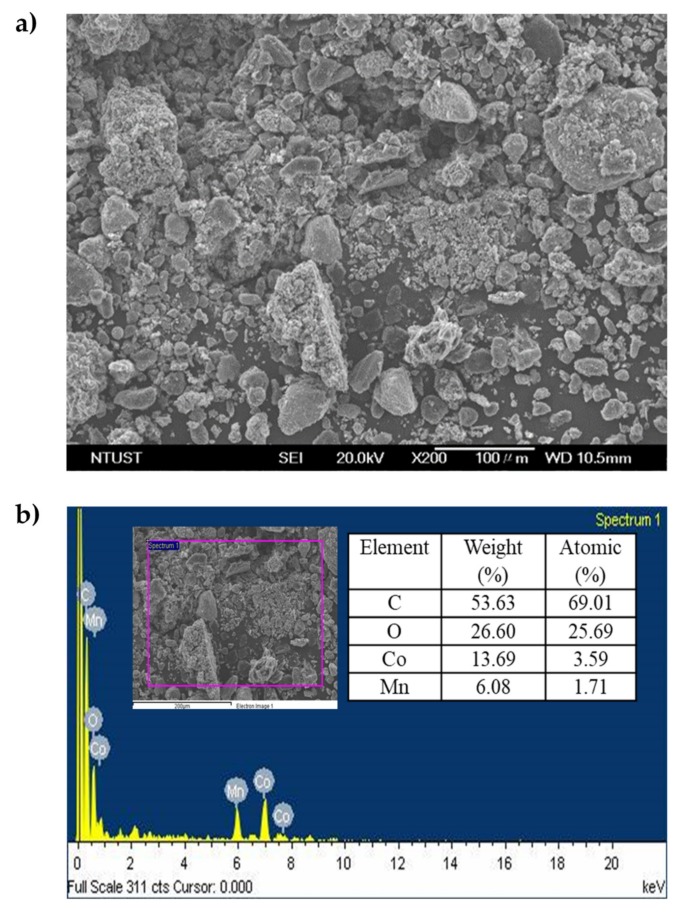
(**a**) FESEM image and (**b**) EDS result of cathode powder of spent LIBs.

**Figure 5:**
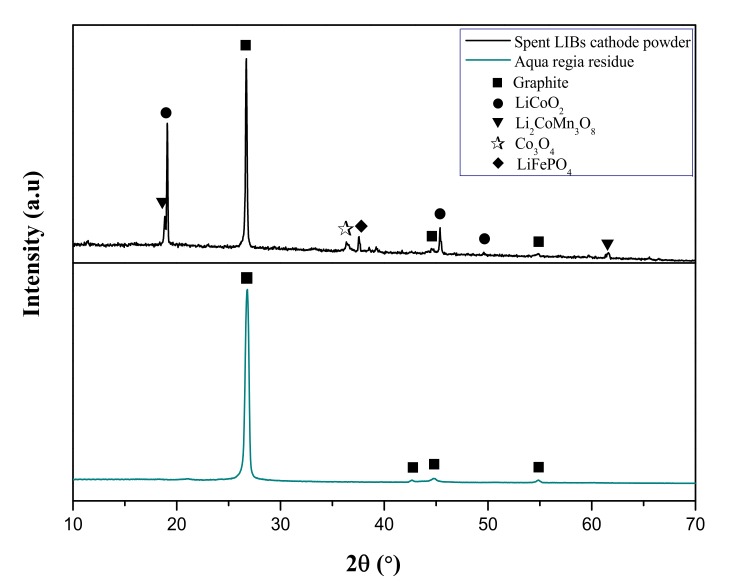
XRD spectra of spent LIBs’ cathode powder and residue of aqua regia digestion.

The spent LIBs’ powder was obtained from Yen-Long Renewable Technology Co., Ltd. (Kaohsiung, Taiwan). It was pretreated by sieving through 140 mesh to retain particle sizes less than 105 μm. The surface morphology of the cathode powder of spent LIBs was examined by field-emission scanning electron microscope with an energy dispersive spectrometer (FESEM-EDS, JSM−6500F, JEOL, Tokyo, Japan) as shown in Figure 4a. The spent LIBs’ powder had irregular shapes, and had sizes ranging from 10 to 100 μm. The elemental composition based on the EDS results can be seen in Figure 4b, which confirmed that spent LIBs contained C from the anode powder, and Co and Mn from the cathode powder. However, Li could not be detected in EDS analysis due to its low atomic number and weight. The X-ray photoelectron spectroscopy (XPS, Thermo Fischer Scientific, VG ESCALAB 250, Waltham, MA, U.S.) analysis was conducted to assess the chemical composition of spent LIBs and the leaching residue. The XPS analysis was carried out with microfocused monochromatized Al Kα radiation hυ = 1200 eV, X-ray gun at 15 kV and 200 W, and beam size 650–120 μm. The binding energy scale was calibrated from the carbon contamination using C 1s peak at 284.5 eV. The aqua regia digestion method (NIEA S321.63B, Taipei, Taiwan) was used to dissolve all of the metals in the spent LIBs’ powder, the solution was then analyzed in inductively coupled plasma-atomic emission spectrometry (ICP-AES, JY−2000, Horiba, Tokyo, Japan) for total metal content. X-ray powder diffraction (XRD, D2 PHASER, Bruker, Karlsruhe, Germany) was also used to characterize the spent LIBs’ powder (Figure 5). It revealed that spent LIBs’ powder was the mixture of the anode, which is mostly graphite (C with COD 9000046), and several kinds of compounds used as the cathode, including lithium cobalt oxide (LiCoO_2_ with PDF 44–0145), lithium cobalt-manganese oxide (Li_2_CoMn_3_O_8_ with PDF 48–0261), cobalt oxide (Co_3_O_4_ with COD 5910031), and lithium iron phosphate (LiFePO_4_ with COD 1011090). The characteristics of leaching residue of aqua regia digestion can be found in Figure 5. The XRD analysis demonstrated that all of the metal hydroxide compounds of the sample were leached out completely.

The SWE of Co, Li, and Mn from spent LIBs were conducted in a 200 mL cylindrical stainless-steel batch reactor (temperature limit: 100–300 °C, pressure range: 0–100 bar) equipped with a magnetic stirrer, thermocouple connected to the electric heater as well as the temperature controller, pressure gauge, and nitrogen gas tube [13]. A solution at 10 g/L of spent LIBs’ powder to leaching agent was placed into the SWE glass chamber and put into the SWE reactor. The reactor was closed and sealed with its cap using M8 screws. The initial pressure was set 10 bar by adding N_2_ gas into the SWE reactor to maintain the leaching process in the liquid phase. It was stirred at 300 rpm and heated to the desired temperature (100, 125, or 150 °C) and kept there for 30 min of leaching time. After cooling to room temperature, the leaching mixture was filtered. The metal content in the leaching solution was analyzed using ICP-AES while the leaching residue was dried at 50 °C in a drying oven for 3 days and characterized. The experiments were carried out in triplicate and the average value was recorded. Eq. 3.1 was used to calculate the leaching efficiency of each metal, in which C_s_ is the concentration of metal in the leaching solution and C_t_ is the total concentration of metal in spent LIBs’ powder as determined by aqua regia digestion shown in Table 3. Cobalt has the highest concentration in the cathode powder (51.34%), followed by Mn (28.12%), Li (7.69%), and other elements including Ni, Al, Fe, and Cu. These results were in agreement with the literature that Co is the most abundant component in LIBs [31].

(1) $$\%\;\mathrm{extraction}=\frac{C_{s}}{C_{t}}\;\times \;100\%$$

## 4. Conclusions

The leaching of valuable metals from spent LIBs’ cathode powder using SWE was investigated, and the results showed that SWE was more effective and efficient in leaching of Co, Li, and Mn than the conventional method. The acid concentration played a key role in the leaching efficiency of spent LIBs using SWE. Ascorbic acid yielded better leaching efficiency than HCl, since it can act as an acidic leaching agent and a stronger reducing agent, while HCl performed mainly as an acidic leaching agent. The redox reactions at the LIBs’ cathode powder surfaces were examined by XPS, which provided solid evidence of the reduction of Co(III) to Co(II) by HCl to a limited extent, and a complete reaction by ascorbic acid. Higher temperatures of SWE using HCl resulted in higher leaching efficiency of valuable metals. However, temperatures >100 °C of SWE using ascorbic acid resulted in lower efficiency owing to decomposition reactions of ascorbic acid under subcritical conditions. Complete leaching of Co, Li, and Mn from cathode powder of spent LIBs were obtained by SWE using 0.5 M of ascorbic acid at 100 °C with 10 g/L solid/liquid ratio, within 30 min. In summary, the SWE process showed the advantages of higher efficiency, shorter reaction time, and lower acid concentrations required. It has very good potential as an alternative process for spent LIBs recycling.

## Figures and Tables

**Table 1:** Redox reaction of cobalt involved in spent lithium-ion batteries (LIBs) leaching.

Leaching by HCl		Leaching by Ascorbic Acid	
Reaction	E°/V *	Reaction	E°/V *
Co^3+^ + e^−^ → Co^2+^	1.92 ^a^	Co^3+^ + e^−^ → Co^2+^	1.92 ^a^
Cl_2(g)_ + 2 e^−^ → 2 Cl^−^	1.36 ^a^	DAsc + 2e^−^ + H^+^ → HAsc^−^	0.28 ^b^
		DAsc + 2e^−^ + 2H^+^ → H_2_Asc	0.39 ^b^
2 Cl^−^ + 2 Co^3+^ → 2 Co^2+^ + Cl_2(g)_	1.24	HAsc^−^ + 2 Co^3+^ → DAsc + 2Co^2+^ + H^+^	1.78
		H_2_Asc + 2 Co^3+^ → DAsc + 2Co^2+^ + 2H^+^	1.73

* selected standard electrode potentials in aqueous solutions at 25 °C in V vs. NHE; ^a^ referred to [22]; ^b^ referred to [23].

**Table 2:** XPS analysis of surface compositions of spent LIBs’ powder and leaching residue.

Peak	Spent LIBs		Leaching Residue HCl		Leaching Residue AA		Assignment
	BE (eV)	Ratio	BE (eV)	Ratio	BE (eV)	Ratio	
Co 2p	780.28	1.000	780.32	1.000	n.d.	n.d.	Co 2p_3/2_: Co(III)
	782.36	1.141	781.82	0.550	n.d.	n.d.	Co 2p_3/2_: Co (II)

**Table 3:** Metal compositions of cathode powder from spent LIBs.

Component	Content (mg/g)	Mass Fraction (%) *
Co	188.93 ± 3.01	51.34
Mn	103.46 ± 2.09	28.12
Li	28.31 ± 0.36	7.69
Ni	17.79 ± 0.11	4.83
Al	12.07 ± 0.07	3.28
Fe	9.27 ± 0.25	2.52
Cu	8.14 ± 0.06	2.21

* percentage among dissolved metals.

## Supplementary Materials

The following are available online. Figure S1: Kinetic plot of Co extraction at different temperature using 0.5 M HCl with 10 g/L of solid to liquid ratio, Figure S2: Kinetic plot of Li extraction at different temperatures using 0.5 M HCl with 10 g/L of solid to liquid ratio, Figure S3: Kinetic plot of Mn extraction at different temperature using 0.5 M HCl with 10 g/L of solid to liquid ratio, Figure S4: Kinetic plot of Co extraction at different temperatures using 0.5 M ascorbic acid with 10 g/L of solid to liquid ratio, Figure S5: Kinetic plot of Li extraction at different temperatures using 0.5 M ascorbic acid with 10 g/L of solid to liquid ratio, Figure S6: Kinetic plot of Mn extraction at different temperatures using 0.5 M ascorbic acid with 10 g/L of solid to liquid ratio, Figure S7: XRD spectra of leaching residues of ascorbic acid and HCl after subject to SWE, Table S1: Kinetic parameters of Co extraction with 0.5 M HCl, Table S2: Kinetic parameters of Li extraction with 0.5 M HCl, Table S3: Kinetic parameters of Mn extraction with 0.5 M HCl, Table S4: Kinetic parameters of Co extraction with 0.5 M ascorbic acid, Table S5: Kinetic parameters of Li extraction with 0.5 M ascorbic acid, Table S6: Kinetic parameters of Mn extraction with 0.5 M ascorbic acid, Table S7: Activation energy using Arrhenius equation for leaching Co, Li, and Mn from spent lithium ion batteries in 0.5 M of HCl and ascorbic acid.
