# Supplementary material for: Subcritical Water Extraction of Valuable Metals from Spent Lithium-Ion Batteries
Source: Molecules. 2020 May 6;25(9):2166. doi: 10.3390/molecules25092166 (PMC7248760; doi:10.3390/molecules25092166)
Supplement: Supplementary file 1 [file molecules-25-02166-s001.pdf]

molecules

Supplementary Materials

Subcritical Water Extraction of Valuable Metals from Spent Lithium-ion Batteries

Jenni Lie, Stefani Tanda and Jhy-Chern Liu *

Department of Chemical Engineering, National Taiwan University of Science and Technology, 43 Keelung Road, Section 4, Taipei 106, Taiwan
* Correspondence: liu1958@mail.ntust.edu.tw

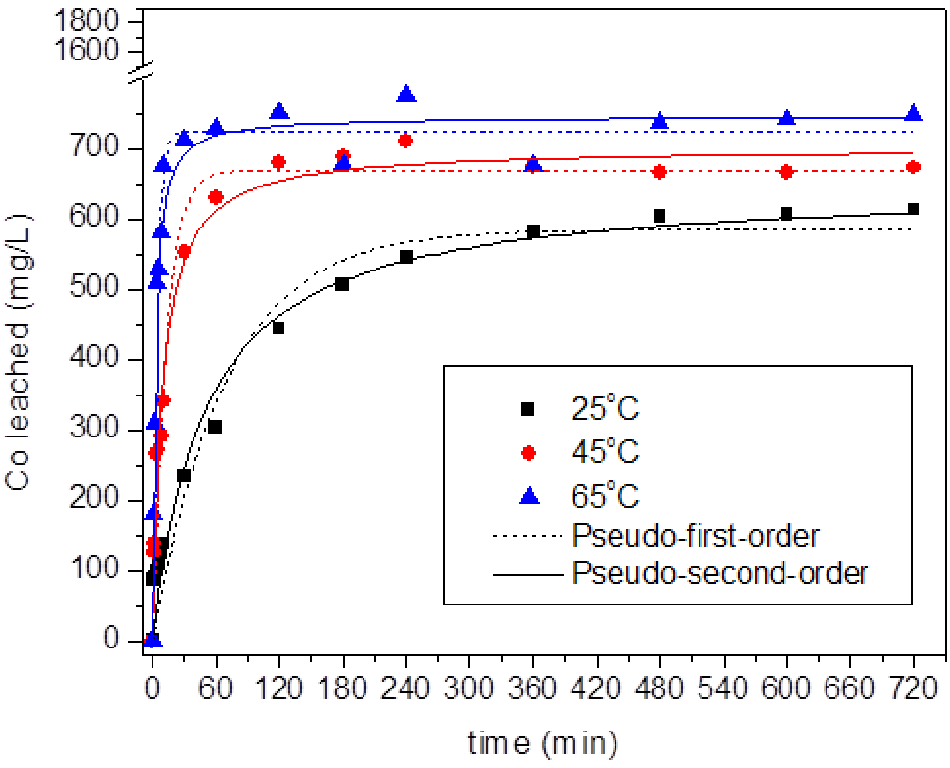

Figure S1. Kinetic plot of Co extraction at different temperature using 0.5 M HCl with 10 g/L of solid to liquid ratio.

Table S1. Kinetic parameters of Co extraction with 0.5 M HCl.

Co	Pseudo-First-Order			Pseudo-Second-Order		
**Temperature**	**Ce**	**k_1**	**R^2**	**Ce**	**k_2**	**R^2**
**25ºC**	584.59	0.01	0.955	648.68	3.19E-05	0.974
**45ºC**	668.74	0.08	0.974	701.36	1.62E-04	0.985
**65ºC**	723.83	0.26	0.978	745.71	5.80E-04	0.973

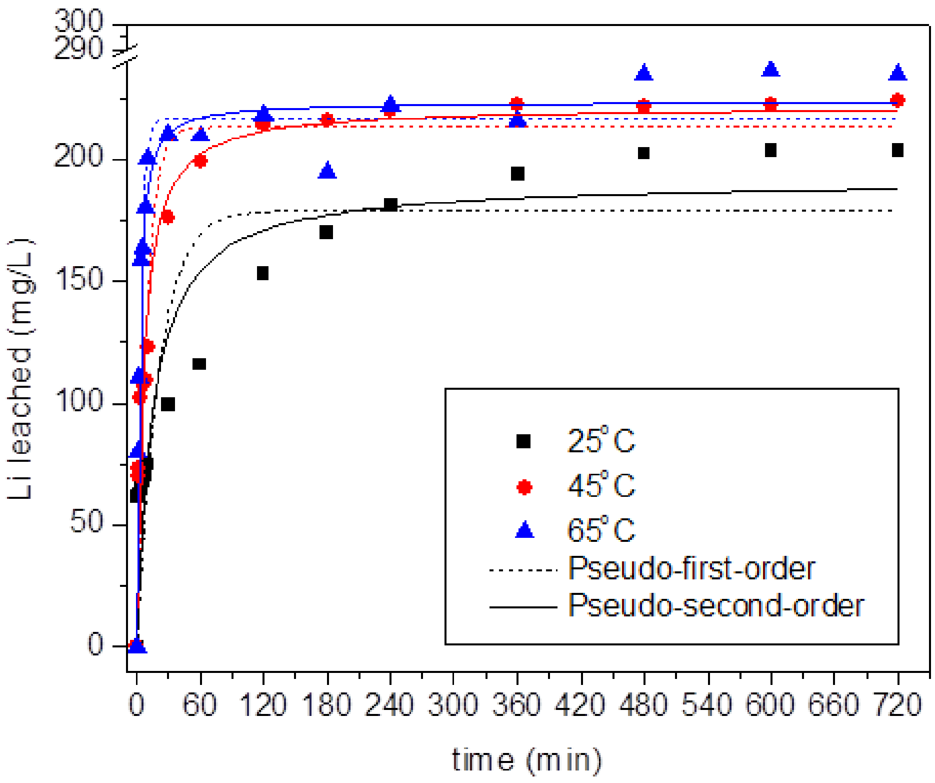

Figure S2. Kinetic plot of Li extraction at different temperatures using 0.5 M HCl with 10 g/L of solid to liquid ratio.

Table S2. Kinetic parameters of Li extraction with 0.5 M HCl.

**Li**	**Pseudo-First-Order**			**Pseudo-Second-Order**		
**Temperature**	**Ce**	**k_1**	**R^2**	**Ce**	**k_2**	**R^2**
**25°C**	178.98	0.04	0.774	191.45	3.60E-04	0.864
**45°C**	213.29	0.11	0.916	221.86	7.67E-04	0.962
**65°C**	216.83	0.31	0.949	223.68	2.41E-03	0.974

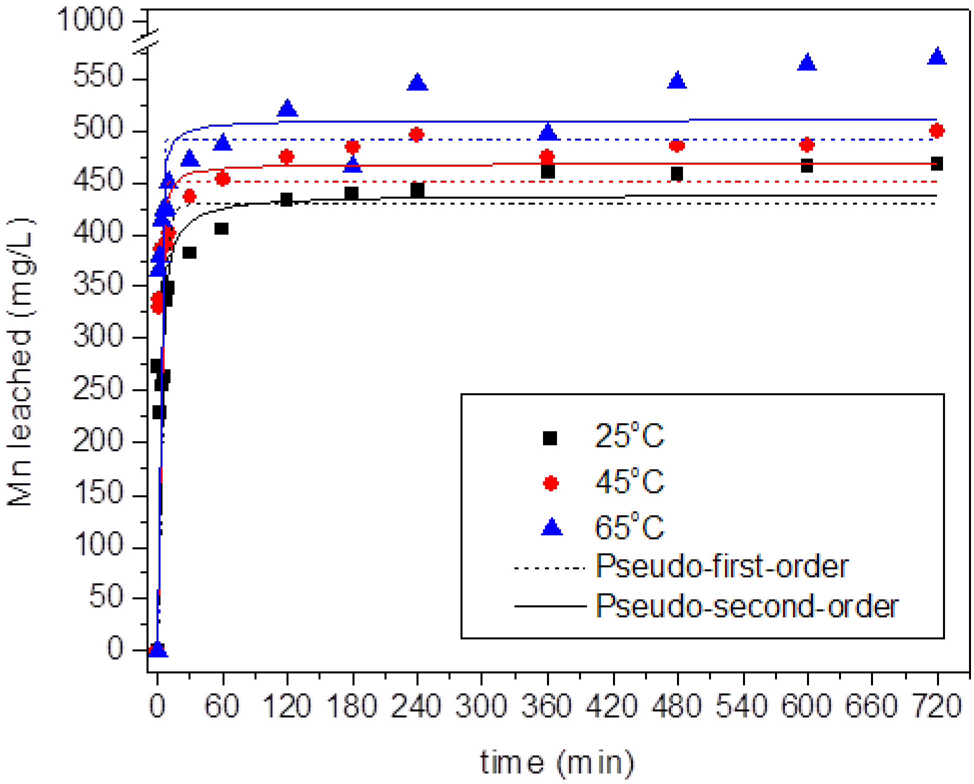

Figure S3. Kinetic plot of Mn extraction at different temperature using 0.5 M HCl with 10 g/L of solid to liquid ratio.

Table S3. Kinetic parameters of Mn extraction with 0.5 M HCl.

Mn	Pseudo-First-Order			Pseudo-Second-Order		
**Temperature**	**Ce**	**k_1**	**R^2**	**Ce**	**k_2**	**R^2**
**25°C**	429.08	0.26	0.770	438.72	1.18E-03	0.882
**45°C**	450.44	0.97	0.872	468.60	3.13E-03	0.950
**65°C**	490.93	1.03	0.844	510.60	3.04E-03	0.919

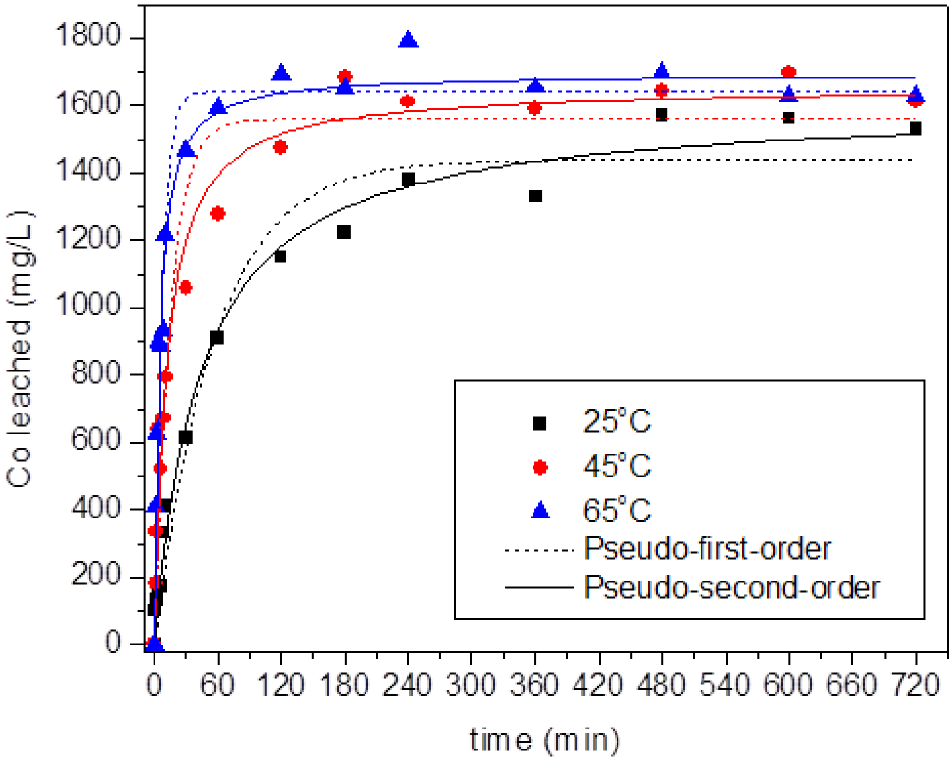

Figure S4. Kinetic plot of Co extraction at different temperatures using 0.5 M ascorbic acid with 10 g/L of solid to liquid ratio.

Table S4. Kinetic parameters of Co extraction with 0.5 M ascorbic acid.

**Co**	**Pseudo-First-Order**			**Pseudo-Second-Order**		
**Temperature**	**Ce**	k_1	R^2	**Ce**	k_2	R^2
**25ºC**	1438.95	0.02	0.967	1604.72	1.45E-05	0.988
**45ºC**	1559.83	0.07	0.938	1656.69	5.39E-05	0.977
**65ºC**	1640.94	0.15	0.949	1695.69	1.38E-04	0.979

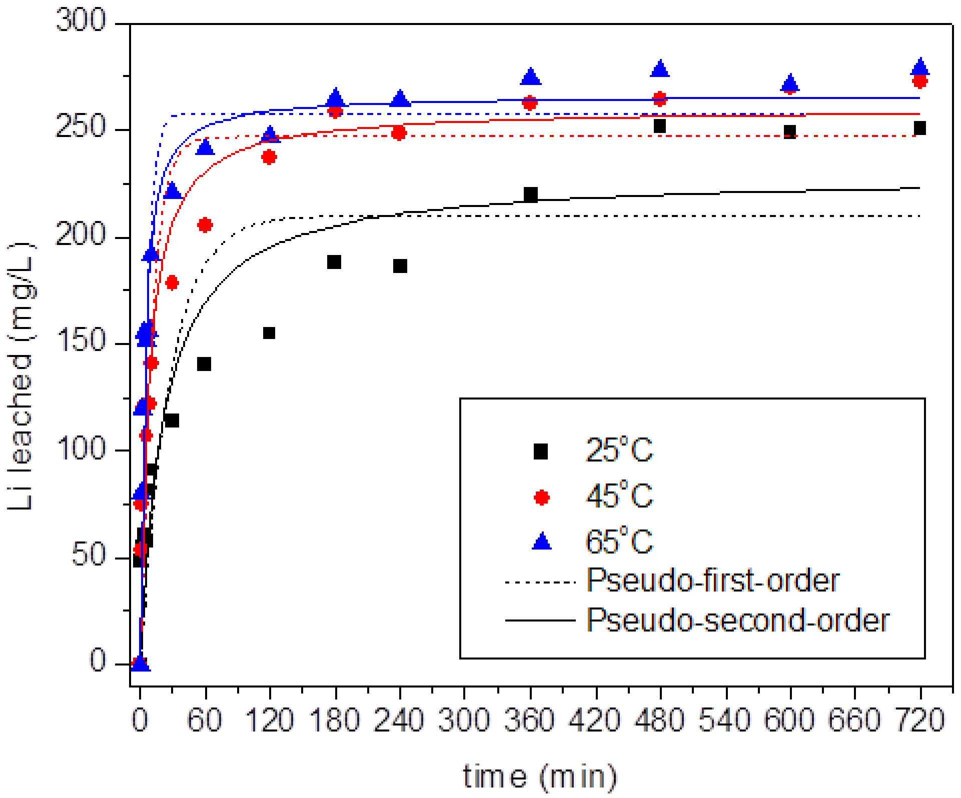

Figure S5. Kinetic plot of Li extraction at different temperatures using 0.5 M ascorbic acid with 10 g/L of solid to liquid ratio.

Table S5. Kinetic parameters of Li extraction with 0.5 M ascorbic acid.

**Li**	**Pseudo-First-Order**			**Pseudo-Second-Order**		
**Temperature**	**Ce**	**k_1**	**R^2**	**Ce**	**k_2**	**R^2**
**25°C**	209.81	0.04	0.814	229.06	2.09E-04	0.892
**45°C**	246.77	0.10	0.904	260.32	4.99E-04	0.961
**65°C**	257.78	0.17	0.904	266.45	1.05E-03	0.965

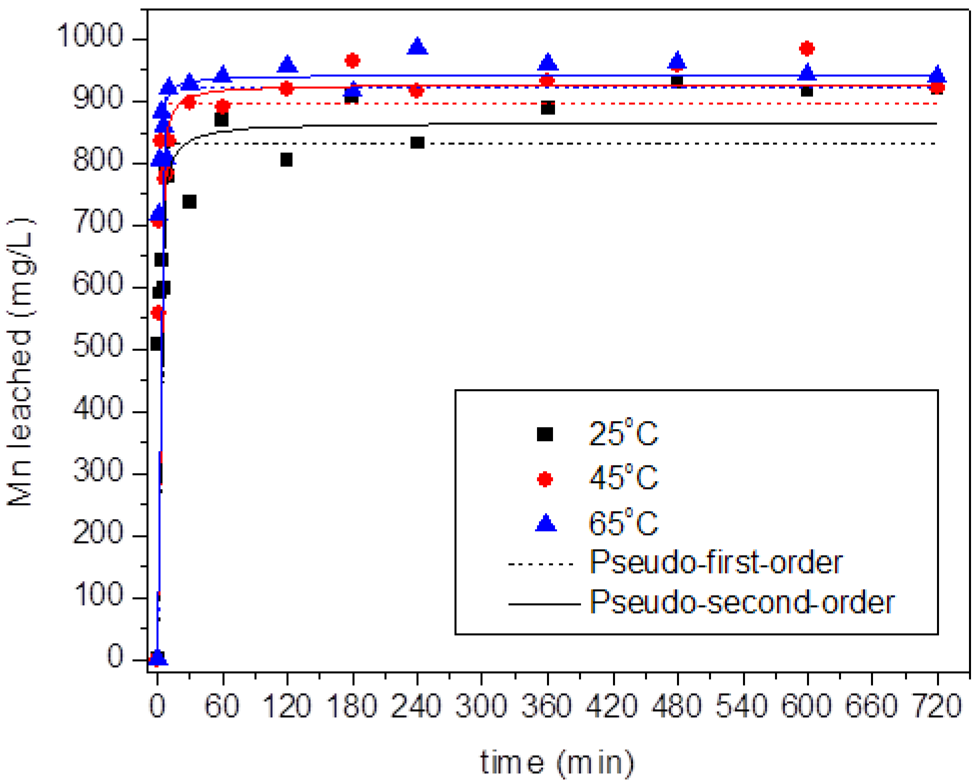

Figure S6. Kinetic plot of Mn extraction at different temperatures using 0.5 M ascorbic acid with 10 g/L of solid to liquid ratio.

Table S6. Kinetic parameters of Mn extraction with 0.5 M ascorbic acid.

**Mn**	**Pseudo-First-Order**			**Pseudo-Second-Order**		
**Temperature**	**Ce**	**k_1**	**R^2**	**Ce**	**k_2**	**R^2**
**25°C**	832.06	0.60	0.842	866.67	1.10E-03	0.928
**45°C**	896.81	0.85	0.935	928.05	1.54E-03	0.977
**65°C**	922.00	1.37	0.959	942.51	3.07E-03	0.981

Table S7. Activation energy using Arrhenius equation for leaching Co, Li, and Mn from spent lithium ion batteries in 0.5 M of HCl and ascorbic acid.

**Element**	**Ea (kJ/mol)**	
	**HCl**	**Ascorbic Acid**
Co	60.823	47.191
Li	39.590	33.928
Mn	20.178	21.229

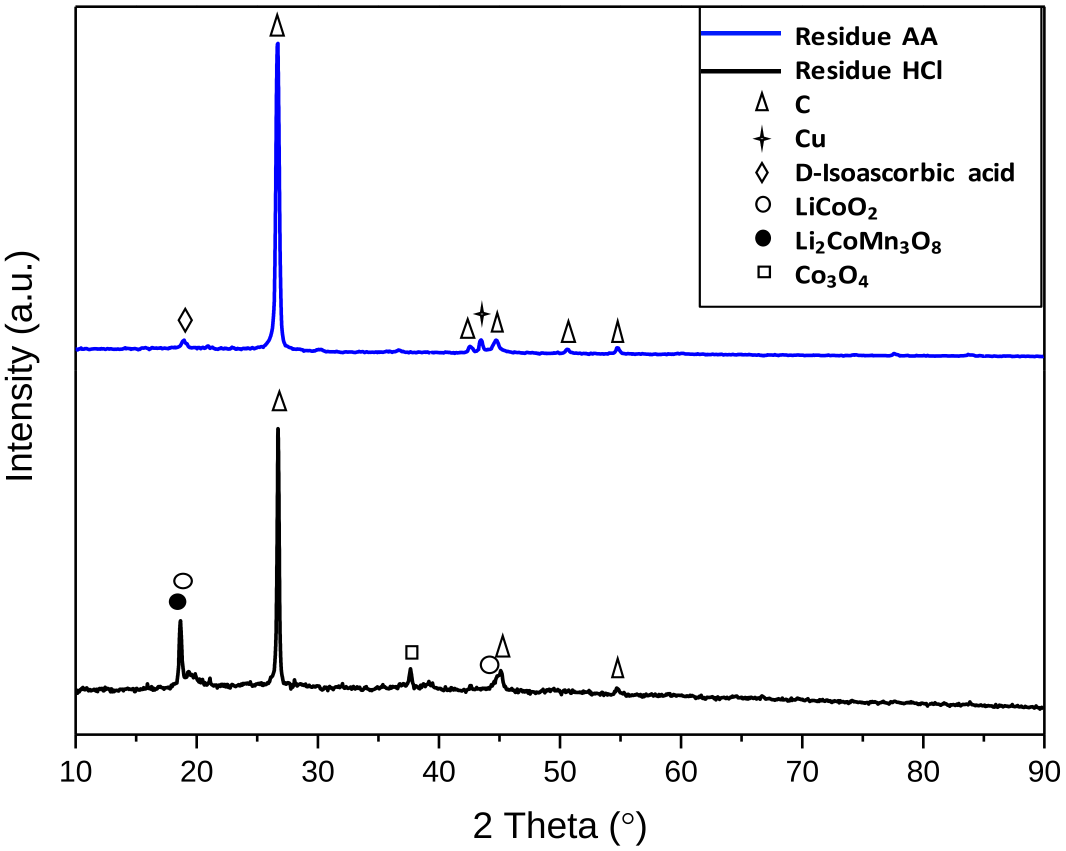

Figure S7. XRD spectra of leaching residues of ascorbic acid and HCl after subject to SWE.
